# Metatranscriptomics as a tool to identify fungal species and subspecies in mixed communities – a proof of concept under laboratory conditions

**DOI:** 10.1186/s43008-019-0012-8

**Published:** 2019-08-08

**Authors:** Vanesa R. Marcelino, Laszlo Irinyi, John-Sebastian Eden, Wieland Meyer, Edward C. Holmes, Tania C. Sorrell

**Affiliations:** 1 Marie Bashir Institute for Infectious Diseases and Biosecurity and Faculty of Medicine and Health, Sydney Medical School, Westmead Clinical School, The University of Sydney, Sydney, NSW 2006 Australia; 2 Molecular Mycology Research Laboratory, Centre for Infectious Diseases and Microbiology, Westmead Institute for Medical Research, Westmead, NSW 2145 Australia; 3 Westmead Hospital (Research and Education Network), Westmead, NSW 2145 Australia; 4 School of Life & Environmental Sciences, Charles Perkins Centre, The University of Sydney, Sydney, NSW 2006 Australia

**Keywords:** Fungi, Meta-transcriptomics, Metagenomics, Microbiome

## Abstract

High-throughput sequencing (HTS) enables the generation of large amounts of genome sequence data at a reasonable cost. Organisms in mixed microbial communities can now be sequenced and identified in a culture-independent way, usually using amplicon sequencing of a DNA barcode. Bulk RNA-seq (metatranscriptomics) has several advantages over DNA-based amplicon sequencing: it is less susceptible to amplification biases, it captures only living organisms, and it enables a larger set of genes to be used for taxonomic identification. Using a model mock community comprising 17 fungal isolates, we evaluated whether metatranscriptomics can accurately identify fungal species and subspecies in mixed communities. Overall, 72.9% of the RNA transcripts were classified, from which the vast majority (99.5%) were correctly identified at the species level. Of the 15 species sequenced, 13 were retrieved and identified correctly. We also detected strain-level variation within the *Cryptococcus* species complexes: 99.3% of transcripts assigned to *Cryptococcus* were classified as one of the four strains used in the mock community. Laboratory contaminants and/or misclassifications were diverse, but represented only 0.44% of the transcripts. Hence, these results show that it is possible to obtain accurate species- and strain-level fungal identification from metatranscriptome data as long as taxa identified at low abundance are discarded to avoid false-positives derived from contamination or misclassifications. This study highlights both the advantages and current challenges in the application of metatranscriptomics in clinical mycology and ecological studies.

**Electronic supplementary material:**

The online version of this article (10.1186/s43008-019-0012-8) contains supplementary material, which is available to authorized users.

## INTRODUCTION

Microscopic fungal species, such as yeasts and some filamentous fungi, do not live in isolation, and are most commonly found within mixed microbial communities containing multiple species and strains. Assessing the diversity of fungi in mixed communities is important because different fungal taxa may exhibit distinctive phenotypes, and consequently may have different pathogenicity or functional roles. For example, in the rhizosphere, changes in fungal community composition have been associated with shifts in nutrient cycling (Hannula et al. 2017). Humans also harbor, or are exposed to, a diverse fungal community that provides a source of opportunistic pathogens (Bandara et al. 2019; Huffnagle and Noverr 2013; Seed 2014). Strain-level fungal diversity may influence therapeutic responsiveness and needs further investigation. Although it is typically assumed that invasive fungal infections are caused by a single strain, multiple *Candida* strains have been observed during the course of a single episode of infection (Soll et al. 1988). Furthermore, nearly 20% of patients with cryptococcosis are infected by multiple strains with different phenotypes and virulence traits (Desnos-Ollivier et al. 2015; Desnos-Ollivier et al. 2010).

Despite its importance, fungal taxonomic diversity is poorly characterized. From over two million fungal species estimated to exist, less than 8% have been described (Hawksworth and Lucking 2017). Even well-known fungal species are often overlooked during routine diagnostic procedures, surveillance and biodiversity surveys (Brown et al. 2012; Enaud et al. 2018; Yahr et al. 2016). This is in part due to challenges in the detection and classification of these organisms, especially microscopic and cryptic species, such as the etiologic agents of cryptococcosis. Currently, two species complexes are recognized: *Cryptococcus neoformans* and *Cryptococcus gattii* (Kwon-Chung et al. 2002). Seven major haploid lineages are found within these two species complexes (*C. neoformans* species complex: VNI, VNII, VNIV, and *C. gattii* species complex: VGI, VGII, VGIII and VGIV) and their recognition as distinct biological species has been debated (Hagen et al. 2015; Kwon-Chung et al. 2017; Ngamskulrungroj et al. 2009). Being able to distinguish closely-related lineages is important because their phenotype, virulence and ecophysiology can vary substantially. For example, the closely related laboratory strains JEC21 and B-3501 of *C. neoformans* var. *neoformans* (VNIV) are 99.5% identical at the genomic sequence level but differ substantially in thermotolerance and virulence (Loftus et al. 2005). Likewise, different virulence and antifungal tolerance traits were observed within lineages of *C. gattii* VGIII (Firacative et al. 2016).

The introduction of high-throughput sequencing (HTS) marked a new era in mycological research, where the vast diversity of fungi can be studied without the need for culturing (Nilsson et al. 2019). To date, amplicon sequencing of genetic markers (metabarcoding) has been the most popular HTS approach to identify fungal species in mixed communities. Despite its indisputable utility, metabarcoding surveys are affected by PCR amplification biases, and even abundant species can go undetected due to primer mismatch (Marcelino and Verbruggen 2016; Nilsson et al. 2019; Tedersoo et al. 2015). In addition, DNA fragments from dead organisms inflate biodiversity estimates in metabarcoding surveys (Carini et al. 2016). Stool samples, for instance, naturally contain food-derived DNA, which cannot be distinguished from the genetic material of the resident gut microbiota when using DNA-based methods. These challenges can be circumvented by directly sequencing actively transcribed genes via RNA-Seq (Wang et al. 2009), hence avoiding the amplification step, and obtaining an unbiased characterization of the living microbial community. Metatranscriptomics has been used to identify RNA viruses in a range of animal samples (Shi et al. 2016; Shi et al. 2017; Wille et al. 2018; Zhang et al. 2018) and to characterize the functional profile of microbial communities (Bashiardes et al. 2016; Kuske et al. 2015). Studies applying metatranscriptomics to mycorrhizal communities have provided valuable insights into the functional roles of fungi in these symbiotic systems (Gonzalez et al. 2018; Liao et al. 2014). However, links between functional and species-level taxonomy have been infrequently sought, likely because fungal identification from metatranscriptome data is considered unreliable below the phylum level (Nilsson et al. 2019). It is therefore currently unknown whether it is feasible to use metatranscriptomics to identify fungi at the species and subspecies level within a mixed community. Challenges can be expected at both the molecular (e.g. RNA isolation and sequencing) and computational levels (e.g. wrong taxonomic assignments and lack of reference data for species identification). This information is fundamental to the investigation of the potential of metatranscriptomics in diagnostics and ecological studies.

Herein, we evaluated the utility of metatranscriptomics as a tool for the simultaneous identification of fungal species using a defined mock community created under laboratory conditions. We focused on the molecular aspects of metatranscriptome sequencing, and therefore created a proof-of-concept data set containing 15 species of Ascomycetes and Basidiomycetes for which draft or complete genome sequences were available. In addition, we investigated whether strains belonging to sister species, such as the *C. neoformans* and *C. gattii* species complexes, could be identified correctly using metatranscriptomics. Rather than focusing on genetic markers, we sought to classify fungal species using the information from all expressed genes, using the totality of NCBI’s nucleotide collection as a reference database. This study paves the way to apply state-of-the art techniques in fungal biodiversity surveys and clinical diagnostics.

## METHODS

A defined fungal community was constructed from 17 isolates, including 15 fungal species and three strains of the *C. neoformans* species complex in addition to one strain of *C. gattii* (Table 1). Fungal strains were obtained from the Westmead Mycology Culture Collection, and were originally derived from clinical isolates, environmental strains or laboratory lineages (Additional file 1: Table S1). As our goal was to obtain a proof-of-concept of the molecular aspects of metatranscriptome sequencing, we only used fungal species containing complete or draft genome sequences in the NCBI RefSeq database (Pruitt et al. 2007). This avoids analytic complexities due to the lack of reference sequence data (see Discussion). Strains were cultured on Sabouraud agar at 27 °C for 72 h. A sweep of colonies was made with a disposable inoculating loop and dispersed in Phosphate-Buffered Saline buffer (PBS). Fungal cells were quantified in a Neubauer chamber and their concentration adjusted such that the fungal mixture contained equal concentrations of each species (10^8^ cells/mL). RNA was isolated with the RNeasy Plus kit (Qiagen), following the manufacture’s protocol, with an initial freeze-thaw step in liquid nitrogen to disrupt fungal cells. The quantity and quality of the RNA extract was determined with the Nanodrop Spectrophotometer (Thermo Scientific) and the Agilent 2200 TapeStation. As some residual DNA was detected, the RNA extract was further treated with DNase I (Qiagen). Ribosomal depletion (Ribo-Zero Gold technology), library preparation and sequencing (Illumina HiSeq HT, 125 bp Paired End) were performed by the Australian Genomics Research Facility. The raw sequence data were deposited in the NCBI Short Read Archive (accession PRJNA521097).

**Table 1:** Species and strains used to construct a mock fungal community for metatranscriptome sequencing

Fungal species	Strain number
*Candida albicans*	WM 229
*Candida auris*	WM 17.110
*Candida glabrata*	WM 13.101
*Candida dubliniensis*	WM 606
*Candida orthopsilosis*	WM 03.136
*Candida tropicalis*	WM 17.08
*Clavispora lusitaniae* (former *Candida lusitaniae*)	WM 14.04
*Cryptococcus gattii* (VGI)	WM 276
*Cryptococcus neoformans* var. *grubii* (VNI)	H99 GC (H99)
*Cryptococcus neoformans* var*. neoformans* (VNIV)	WM 01.133 (B-3501A)
*Cryptococcus neoformans* var*. neoformans* (VNIV)	WM 01.127 (JEC21)
*Debaryomyces hansenii*	WM 36
*Pichia kudriavzevii* (former *Candida krusei*)	WM 14
*Pichia membranifaciens*	WM 46
*Saccharomyces cerevisiae*	WM 318
*Schizosaccharomyces pombe*	WM 72
*Yarrowia lipolytica*	WM 63

Sequence reads containing more than five ambiguous positions or with average quality scores ≤25 were filtered from the data set using prinseq-lite v.0.20.4 (Schmieder and Edwards 2011) with the options -ns_max_n 5 -min_qual_mean 25 -out_format 3. Assembly of sequence reads into contigs was performed with Trinity v.2.5.1 (Grabherr et al. 2011). Contigs were mapped to the NCBI nucleotide collection using KMA v1.1.7 (Clausen et al. 2018), a novel approach that has proven to be more accurate than other mapping software. Prior to mapping, NCBI’s taxonomic identifier codes (taxids) were appended to each sequence record in the nucleotide collection, and the reference database was indexed using KMA’s options -NI -Sparse TG. Contigs were then mapped to the indexed database with the options -mem_mode -and -apm f. Matches to the reference database with low support (i.e. coverage < 20 and depth < 0.05) were excluded from the analyses. The species-level taxonomic classifications were based on NCBI’s taxonomy identifiers (taxids) to minimize the issue of changing species nomenclature (Federhen 2012). Species names were manually checked in MycoBank (Robert et al. 2013), and the only two discordances observed between the NCBI taxonomy database and MycoBank were *Candida pseudohaemulonis* (which is referred to as *C. pseudohaemulonii* in MycoBank), and *Candida glycerinogenes*, which was not found in MycoBank. The glycerol-producing *C. glycerinogenes* has been described elsewhere using a different spelling (*C. glycerolgenesis -* also not in MycoBank) (Wang et al. 1999), but the *C. glycerinogenes* spelling used here has been used in subsequent publications concerning this species (e.g. Chen et al. 2008; Ji et al. 2016) and is a recognized species name in the NCBI taxonomy database. Subspecies-level classification within the *Cryptococcus neoformans* and *C. gattii* species complexes was made following the ISHAM consensus MLST scheme for the *C. neoformans* and *C. gattii* species complexes (Meyer et al. 2009) and manually examined.

Abundance was estimated at the level of sequence reads and transcripts. For read-level abundances, sequence reads were mapped to transcripts using Bowtie2 v.2.3.3.1 (Langmead and Salzberg 2012) and quantified in Transcripts Per Million (TPM) with RSEM v.1.2.31 (Li and Dewey 2011), using the Trinity pipeline. For transcript-level abundances, the depth values estimated within KMA were used, which is the total number of nucleotides (in each contig) covering the reference sequence divided by the length of the reference sequence. The number and length of assembled contigs for each taxon is likely a better proxy for species abundance than read-level abundances (which are subject to gene expression), and therefore were used for graphic representation and analyses. For simplicity, we refer as ‘abundance’ the transcript-level abundance, unless otherwise stated.

It is possible that species with larger and gene-rich genomes express a greater number of transcripts. To test for this potential correlation, genome sizes and the estimated number of proteins were obtained from the Fungal Genome Size Database (Kullman et al. 2005), Loftus et al. (2005), Munoz et al. (2018) and NCBI’s Genome database (Additional file 1: Table S1). The correlation coefficients between genome size, number of proteins and abundance of transcripts were estimated using Person’s correlation and visualized using the R package *ggpubr* v.0.2 (Kassambara 2017).

## RESULTS

**Fig. 1:**
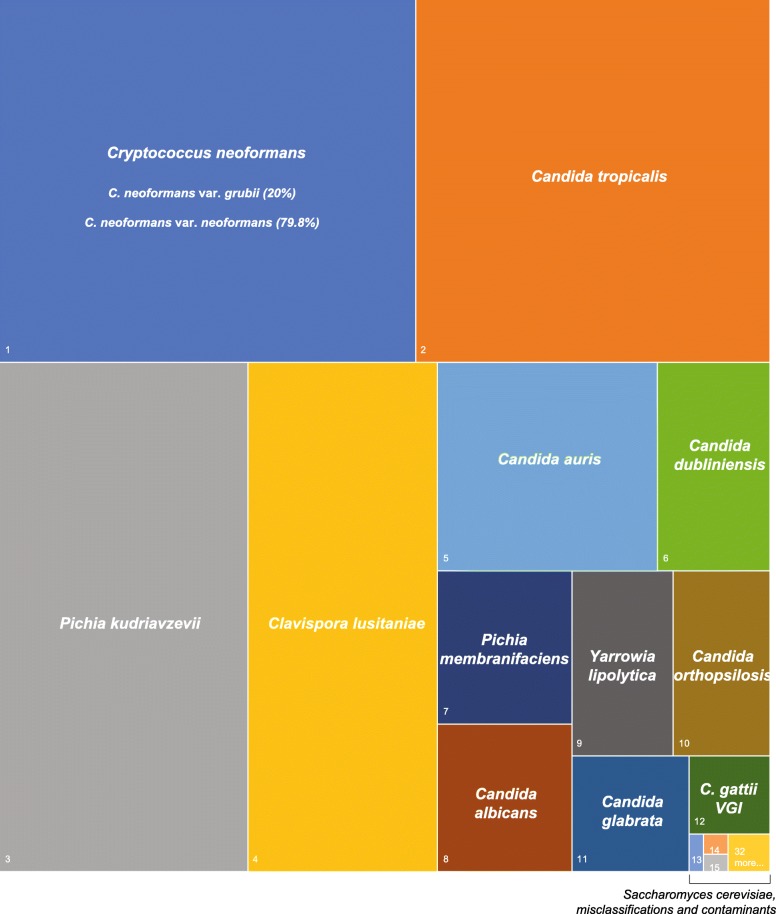
Relative abundance of transcripts assigned to microbial species in the metatranscriptome of the mock community used in this study. The size of the rectangles represents the relative abundance of different species. See Table 2 for a full list of species and more details about their abundance

RNA sequencing yielded a total of 26,558,491 paired end reads, of which 98.3% passed quality control. Overall, 277,404 contigs (transcripts) were obtained, from which 202,219 (72.9%) were classified. The majority of the sequence reads (80.2%) mapped to a classified contig. Of the 15 fungal species sequenced, 13 were retrieved and correctly classified at the species level (Fig. 1, Table 2, Additional file 1: Table S2). The two false-negatives were *Debaryomyces hansenii* and *Schizosaccharomyces pombe*; these may have been misclassified as another fungus or were lost due to cell pooling inaccuracy and/or RNA extraction biases. A small proportion of bacterial transcripts (0.03%) and other eukaryotic microbes (0.4%, including 31 fungi that were not present in the mock community) was also observed (Table 2, Additional file 1: Table S2), which likely represent laboratory contaminants and misclassifications (see Discussion). However, these were present at a consistently lower frequency than true members of the mock community, with the most common – *Candida glycerinogenes* – only present in 0.08% of the transcripts. Some of the transcripts were assigned to entries in GenBank that do not have a species-level classification (e.g. *Candida* sp. and *Pichia* sp*.*). These assignments were considered misclassifications here, although it is possible that the species in our mock community are the correct species-level identity of these GenBank sequences.

**Table 2:** Abundance of reads (TPM) and abundance of transcripts (Depth) per fungal species detected with metatranscriptomics. True members of the mock community – at species level – are shown in bold

Species^a^	TPM (read-level)	Depth (transcript-level)	Relative abundance (transcript-level %)
*Cryptococcus neoformans*	**149,692.28**	**11,049.04**	**22.464**
*Candida tropicalis*	**142,496.47**	**9424.30**	**19.161**
*Pichia kudriavzevii*	**62,133.74**	**9234.05**	**18.774**
*Clavispora lusitaniae*	**57,317.81**	**7107.80**	**14.451**
*Candida auris*	**13,402.41**	**3354.39**	**6.820**
*Candida dubliniensis*	**52,027.94**	**1706.41**	**3.469**
*Pichia membranifaciens*	**10,860.75**	**1498.26**	**3.046**
*Candida albicans*	**42,948.69**	**1441.56**	**2.931**
*Yarrowia lipolytica*	**52,376.03**	**1384.29**	**2.814**
*Candida orthopsilosis*	**44,531.25**	**1308.09**	**2.660**
*Candida glabrata*	**50,404.11**	**992.55**	**2.018**
*Cryptococcus gattii* VGI	**9350.81**	**463.71**	**0.943**
*Candida glycerinogenes*	2345.13	41.51	0.084
*Nakaseomyces delphensis*	12,821.76	35.45	0.072
*Candida parapsilosis*	15,989.78	31.11	0.063
*Candida nivariensis*	1411.36	12.72	0.026
*Kluyveromyces marxianus*	47.70	8.90	0.018
*Torulaspora delbrueckii*	15.01	6.00	0.012
*Kluyveromyces lactis*	9.24	3.93	0.008
*Saccharomyces cerevisiae*	**22.84**	**3.77**	**0.008**
*Eremothecium sinecaudum*	25.93	3.67	0.008
*Pichia cecembensis*	751.23	3.60	0.007
*Lodderomyces elongisporus*	34.30	3.59	0.007
Uncultured *Candida*	885.35	3.13	0.006
*Eremothecium gossypii*	10.13	3.04	0.006
*Naumovozyma dairenensis*	17.37	2.80	0.006
*Suhomyces tanzawaensis*	30.87	2.25	0.005
*Dipodascaceae* sp. LM136	24,286.11	2.16	0.004
*Cyberlindnera jadinii*	12.32	2.04	0.004
*Metschnikowia bicuspidata*	16.02	1.29	0.003
*Brettanomyces naardenensis*	96.13	1.19	0.002
*Pichia norvegensis*	783.06	1.11	0.002
*Debaryomyces fabryi*	22.87	0.92	0.002
*Candida neerlandica*	487.65	0.69	0.001
*Melanotaenium endogenum*	262.12	0.59	0.001
*Pichia kluyveri*	51,814.00	0.55	0.001
*Candida pseudohaemulonis* ^b^	560.14	0.49	0.001
*Candida* sp. (in: *Saccharomycetales*)	330.15	0.49	0.001
*Pichia* sp. 2 TMS-2011	0.00	0.45	0.001
*Cryptococcus neoformans* AD hybrid	0.00	0.44	0.001
*Saccharomycetales* sp. LM594	2.60	0.30	0.001
*Naumovozyma castellii*	4.56	0.29	0.001
*Saccharomyces pastorianus*	0.81	0.26	0.001
*Cryptococcus gattii* VGIII	0.48	0.21	0.000
Other Eukaryotes	936.75	18.83	0.038
Bacteria	423.36	15.79	0.032
Unclassified	198,000.57	8.00	0.016
TOTAL	**1,000,000**	**49,186**	**100**

^a^ Species defined according to the NCBI taxonomy database. Strain numbers may indicate vouchers rather than genetically different lineages. ^b^*Candida pseudohaemulonis* is also referred to as *C. pseudohaemulonii*

Overall, the commonest species detected was *C. neoformans*, which was expected as it comprised three strains in the mock community and therefore was three times more abundant than other fungal species*.* Transcripts belonging to *Candida tropicalis* and *Pichia kudriavzevii* (former *Candida krusei*) – were also common (19.2 and 18.8%, respectively), while *C. albicans*, *C. orthopsilosis* and *C. glabrata* (other causes of candidaemia in humans) were detected at lower abundance (2.0–2.9%). There was no relationship between abundance of transcripts and phylogenetic relatedness. Genomes with low GC content can be overrepresented in metagenomic sequencing (Shakya et al. 2013). Conversely, some of the species detected here in high abundance (*Cryptococcus neoformans* and *Clavispora lusitaniae*) have a higher GC content than most other fungal species (Dujon 2010), suggesting that GC bias is unlikely to affect our results. No correlation between abundance of transcripts and genome size or estimated number of proteins was observed (*p* > 0.05, Additional file 2).

**Fig. 2:**
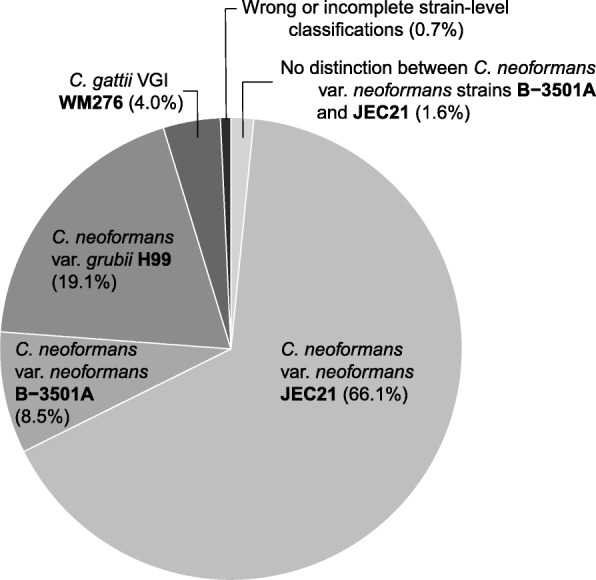
Strain-level classifications of taxa within the *Cryptococcus neoformans* and *C. gattii* species complexes

Molecular type and strain-level variation within the *Cryptococcus neoformans* and *C. gattii* species complexes was also detected, with contigs matching to *C. gattii* VGI WM 276*, C. neoformans* var. *grubii* VNI H99 and *C. neoformans* var. *neoformans* VNIV strains B-3501A and JEC21 (Fig. 2, Additional file 1: Table S3). A proportion of the transcripts (1.6%) matched with equal probability scores to both strains of *C. neoformans* var. *neoformans* (B-3501A and JEC21, Additional file 1: Table S2 and Table S3). From the transcripts classified as *Cryptococcus* spp*.,* 99.3% were classified as one of the four *Cryptococcus* strains (or both B-3501A and JEC21) used in the mock community. It is possible that misclassifications occurred within the strains analyzed. For example, transcripts originally from JEC21 might have been classified as B-3501A and vice versa. As it is not possible to know from which strain the transcripts originated, these possible misclassifications would be undetected.

The total costs for RNA extraction, library preparation and sequencing were AUD $806 (~USD $558). The library preparation was the most expensive process, at AUD $400 per sample.

## DISCUSSION

Our metatranscriptomics approach yielded taxonomic identification of fungi from a defined mock community with high success, while false-positives were detected at far lower abundance. This proof-of-concept study therefore indicates that it is possible to obtain accurate species- and strain- level identifications for fungi from metatranscriptome data, as long as taxa identified at low abundance are removed from the analyses to avoid false-positives derived from contamination or misclassifications.

Taxonomic classification at species and strain levels using metabarcoding and metagenomic data has been considered inaccurate (Nilsson et al. 2019; Sczyrba et al. 2017; Yamamoto et al. 2014), raising the question of how our metatranscriptomics approach succeeded in identifying closely-related fungal strains. Metabarcoding relies on a single or multiple genetic marker(s) (Banchi et al. 2018; e.g. McGuire et al. 2013; Schmidt et al. 2013), which does not contain sufficient phylogenetic information to differentiate some closely related fungal lineages (Balasundaram et al. 2015; Nilsson et al. 2008). Metatranscriptomics, on the other hand, potentially yields data on all expressed coding sequences. Classifications derived from metagenomes are likely to be equally accurate as the ones obtained from metatranscriptomes, except that dead organisms might also be sequenced. Additionally, we used a new alignment method (KMA) that is both highly accurate and fast (Clausen et al. 2018), allowing us to map sequences against the complete NCBI nucleotide collection. Metatranscriptomics is also less susceptible to amplification bias, no information about the community members is required a priori, and it only detects functionally active members of a microbial community. These advantages make metatranscriptomics a promising tool in biodiversity surveys, functional assessments of microbial communities, pathogen detection and biosecurity surveillance (e.g. Kuske et al. 2015; Shi et al. 2016; Wille et al. 2018).

We constructed a model community to establish a proof-of-concept for fungal metatranscriptomics, using only organisms for which draft or complete genome sequences were available and with fresh cultures processed under ideal laboratory conditions. Clearly, routine applications of metatranscriptomics have additional hurdles that should be taken into consideration. First, RNA is more unstable than DNA and quickly degrades unless the sample is frozen or embedded in a stabilizing reagent, which can introduce biases in the study (Reck et al. 2015). Second, the community complexity may affect taxonomic identification, as the number of sequence reads per species is inversely proportional to the number of species in the community. This is especially problematic in communities containing a diverse and abundant bacterial community, which can dominate the metatranscriptome. Finally, the lack of reference sequences is a major issue, especially when working with poorly characterized systems. Considering that complete genome sequences are available for only a fraction of the over 2 million species estimated to exist (Hawksworth and Lucking 2017), it is likely that the entire NCBI nucleotide collection is the most comprehensive database available to identify fungi in metagenomes and metatranscriptomes. Importantly, it is possible to extract informative genes from metatranscriptome data and subsequently perform phylogenomic analyses to identify rare and novel taxa with more certainty (e.g. Shi et al. 2017; Wille et al. 2018; Zhang et al. 2018).

Even though false-positives were present at low abundance, they do pose a challenge in the interpretation of metatranscriptomic and metagenomic data. False-positives generally result from spurious classifications and laboratory contaminants, which may be common in laboratory reagents (Salter et al. 2014). Metatranscriptomics is less sensitive to laboratory contamination than DNA-based metagenomics or metabarcoding, as only living microorganisms are sequenced. Nevertheless, contamination can occur at various stages of the library preparation and is routinely observed in RNA-Seq studies (Quince et al. 2017; Strong et al. 2014). Misclassifications occur because some genome regions are very similar (or identical) across closely-related species, which cannot be differentiated. This might be the case for some false-positives that are closely-related to some of the species used in the mock community, like *Candida parapsilosis* (a false-positive) which is closely related to *Candida orthopsilosis* (true positive). Errors in reference databases can also result in misclassifications. Sequences attributed to incorrectly-classified species are not uncommon in GenBank and result in downstream classification errors (Li et al. 2018). It is also not unusual to find bacterial regions misassembled into eukaryotic genomes (e.g. Koutsovoulos et al. 2016), which can result in sequences from common laboratory contaminants being classified as a eukaryote. Filtering out organisms found in low abundance is an option to reduce the incidence of false-positives in downstream analyses. In this study, filtering organisms for which the abundance of transcripts is lower than 0.1% would eliminate false-positives, at the cost of excluding one true-positive from the analyses (Table 2). The threshold for this abundance-filtering depends on the desired balance between precision and recall. The application of an abundance-filtering step might not be feasible when sequencing depth (per microbial species) is limited. Species present at low abundance will be represented by a small number of transcripts and so are more likely to be misclassified or undetected.

The abundance disparity across species and the fact that we did not retrieve two species from the mock community (*D. hansenii* and *S. pombe*) shows that the method and our experimental design have limitations, even when performed under ideal laboratory conditions. The abundance of transcripts could vary according to genome size, number of coding sequences, and gene expression. However, we found no correlation between the abundance of transcripts and genome size or number of genes (Additional file 2), suggesting that gene expression prevails over gene numbers in defining transcript abundance. Imprecise estimates of cell abundance and RNA extraction biases could also have influenced abundance estimates. It is widely recognized that biases in extracting genetic material from mixed communities affect abundance and diversity estimates (Martin-Laurent et al. 2001), although we are unaware of features of *D. hansenii* and *S. pombe* that could have influenced the extraction of their RNA. Metabarcoding studies have suggested that performing DNA extraction in triplicate minimizes biases for bacteria, but it had no effect in fungal communities (Feinstein et al. 2009). To our knowledge, the effect of RNA extraction bias in metatranscriptomics has yet to be studied. It is possible that competition between fungal species influences gene expression, although we believe that this would have a negligible effect given that the samples were processed immediately after pooling the species together. As a single metatranscriptome library was sequenced, it is difficult to estimate the impact of these potential biases, and more insights may be obtained by analyzing communities with a different composition and different levels of complexity. RNA extraction bias depends on the species composition of the sample (for example, whether some species have harder cell walls that may hinder RNA extraction), potential inhibitors found in the study system and sample processing (including human error). Alternatively, as metagenomics surveys are not affected by gene expression, they might be more appropriate for studies where it is important to quantify species abundance.

Although fungal species and their genes can be confidently identified, it remains challenging to link some genes with particular species using metatranscriptomics. A large portion of fungal genomes are highly similar among species, making it difficult, if not impossible, to infer which species in the community are expressing which genes. Recently, a method was developed to perform species-level functional profiling of metagenome data (Franzosa et al. 2018). This method, however, relies on a reference database of complete genomes that currently contains few fungal representatives, limiting its application in fungal metagenomics. Contrary to metatranscriptomics, metagenomics yields coding and non-coding sequences, which can facilitate linking genes to species if sequencing depth is large enough to assemble large parts of fungal genomes (e.g. Olm et al. 2019).

## CONCLUSIONS

In sum, we show that metatranscriptomics is a viable alternative approach to identify fungal species and subspecies in mixed samples from a model fungal community. The major advantages of metatranscriptomics over other HTS technologies include the selective sequencing of living organisms and the ability to detect a wide range of microorganisms in one step, which has multiple applications in biological research, surveillance and diagnosis. There is an increasing literature reporting that virulence and antimicrobial tolerance traits vary within species (e.g. Firacative et al. 2016; Rizzetto et al. 2013) and that multiple strains or species can co-infect a host (e.g. Desnos-Ollivier et al. 2010; Soll et al. 1988). The high discriminatory power obtained for closely-related lineages of *Cryptococcus* provides a good example of where metatranscriptomics would be valuable in precision medicine, where therapy practices are defined according to strain-specific pathogenicity and drug susceptibility traits. However, it must also be acknowledged that metatranscriptomics has limitations that are common to high-throughput sequencing methods, as it is susceptible to DNA/RNA extraction biases, contamination and misclassifications. These limitations can be minimized if appropriate controls are in place (e.g. abundance filtering before statistical analyses). Besides its application to identify well-known fungal species, metatranscriptomics can help to identify novel functional roles of fungi (e.g. Gonzalez et al. 2018; Liao et al. 2014) and novel species when used within a phylogenomic framework.

## Additional files

Additional file 1: **Table S1.** Metadata of the strains used to construct the mock fungal community, including genome size and protein count. **Table S2.** Mapping results. **Table S3.** Classification of transcripts at subspecies level in the *Cryptococcus gattii* / *C. neoformans* species complex. (XLSX 3006 kb)

Additional file 2: Figure indicating that no correlation between abundance of transcripts (depth) and genome size (A) or protein count (B) can be observed. (PDF 881 kb)

## Data Availability

The raw sequence data were deposited in the NCBI Short Read Archive (accession PRJNA521097).

## Abbreviations

HTS: High-throughput sequencing
MLST: Multilocus sequence typing
RNA-Seq: RNA sequencing

## References

[CR1] Balasundaram SV, Engh IB, Skrede I, Kauserud H (2015). How many DNA markers are needed to reveal cryptic fungal species?. Fungal Biology.

[CR2] Banchi E, Ametrano CG, Stankovic D, Verardo P, Moretti O, Gabrielli F, Lazzarin S, Borney MF, Tassan F, Tretiach M, Pallavicini A, Muggia L (2018). DNA metabarcoding uncovers fungal diversity of mixed airborne samples in Italy. PLoS One.

[CR3] Bandara H, Panduwawala CP, Samaranayake LP (2019). Biodiversity of the human oral mycobiome in health and disease. Oral Diseases.

[CR4] Bashiardes Stavros, Zilberman-Schapira Gili, Elinav Eran (2016). Use of Metatranscriptomics in Microbiome Research. Bioinformatics and Biology Insights.

[CR5] Brown GD, Denning DW, Gow NA, Levitz SM, Netea MG, White TC (2012). Hidden killers: human fungal infections. Science Translational Medicine.

[CR6] Carini P, Marsden PJ, Leff JW, Morgan EE, Strickland MS, Fierer N (2016) Relic DNA is abundant in soil and obscures estimates of soil microbial diversity. Nature Microbiology 2:16242. 10.1038/nmicrobiol.2016.242 10.1038/nmicrobiol.2016.242 27991881

[CR7] Chen X, Fang H, Rao Z, Shen W, Zhuge B, Wang Z, Zhuge J (2008). An efficient genetic transformation method for glycerol producer *Candida glycerinogenes*. Microbiological Research.

[CR8] Clausen P, Aarestrup FM, Lund O (2018). Rapid and precise alignment of raw reads against redundant databases with KMA. BMC Bioinformatics.

[CR9] Desnos-Ollivier M, Patel S, Raoux-Barbot D, Heitman J, Dromer F, French Cryptococcosis Study G (2015). Cryptococcosis serotypes impact outcome and provide evidence of *Cryptococcus neoformans* speciation. MBio.

[CR10] Desnos-Ollivier M, Patel S, Spaulding AR, Charlier C, Garcia-Hermoso D, Nielsen K, Dromer F (2010) Mixed infections and *In Vivo* evolution in the human fungal pathogen *Cryptococcus neoformans*. MBio 1(1). 10.1128/mBio.00091-10 10.1128/mBio.00091-10 PMC2912664 20689742

[CR11] Dujon B (2010). Yeast evolutionary genomics. Nature Reviews. Genetics.

[CR12] Enaud R, Vandenborght LE, Coron N, Bazin T, Prevel R, Schaeverbeke T, Berger P, Fayon M, Lamireau T, Delhaes L (2018). The mycobiome: a neglected component in the microbiota-gut-brain axis. Microorganisms.

[CR13] Federhen S (2012). The NCBI taxonomy database. Nucleic Acids Research.

[CR14] Feinstein LM, Sul WJ, Blackwood CB (2009). Assessment of bias associated with incomplete extraction of microbial DNA from soil. Applied and Environmental Microbiology.

[CR15] Firacative C, Roe CC, Malik R, Ferreira-Paim K, Escandon P, Sykes JE, Castanon-Olivares LR, Contreras-Peres C, Samayoa B, Sorrell TC, Castaneda E, Lockhart SR, Engelthaler DM, Meyer W (2016). MLST and whole-genome-based population analysis of *Cryptococcus gattii* VGIII links clinical, veterinary and environmental strains, and reveals divergent serotype specific sub-populations and distant ancestors. PLoS Neglected Tropical Diseases.

[CR16] Franzosa EA, McIver LJ, Rahnavard G, Thompson LR, Schirmer M, Weingart G, Lipson KS, Knight R, Caporaso JG, Segata N, Huttenhower C (2018). Species-level functional profiling of metagenomes and metatranscriptomes. Nature Methods.

[CR17] Gonzalez E, Pitre FE, Page AP, Marleau J, Guidi Nissim W, St-Arnaud M, Labrecque M, Joly S, Yergeau E, Brereton NJB (2018). Trees, fungi and bacteria: tripartite metatranscriptomics of a root microbiome responding to soil contamination. Microbiome.

[CR18] Grabherr MG, Haas BJ, Yassour M, Levin JZ, Thompson DA, Amit I, Adiconis X, Fan L, Raychowdhury R, Zeng Q, Chen Z, Mauceli E, Hacohen N, Gnirke A, Rhind N, di Palma F, Birren BW, Nusbaum C, Lindblad-Toh K, Friedman N, Regev A (2011). Full-length transcriptome assembly from RNA-Seq data without a reference genome. Nature Biotechnology.

[CR19] Hagen Ferry, Khayhan Kantarawee, Theelen Bart, Kolecka Anna, Polacheck Itzhack, Sionov Edward, Falk Rama, Parnmen Sittiporn, Lumbsch H. Thorsten, Boekhout Teun (2015). Recognition of seven species in the Cryptococcus gattii/Cryptococcus neoformans species complex. Fungal Genetics and Biology.

[CR20] Hannula SE, Morrien E, de Hollander M, van der Putten WH, van Veen JA, de Boer W (2017). Shifts in rhizosphere fungal community during secondary succession following abandonment from agriculture. ISME Journal.

[CR21] Hawksworth DL, Lucking R (2017) Fungal diversity revisited: 2.2 to 3.8 million species. Microbiology Spectrum 5(4). 10.1128/microbiolspec.FUNK-0052-2016 10.1128/microbiolspec.funk-0052-2016 PMC11687528 28752818

[CR22] Huffnagle GB, Noverr MC (2013). The emerging world of the fungal microbiome. Trends in Microbiology.

[CR23] Ji H, Zhuge B, Zong H, Lu X, Fang H, Zhuge J (2016). Role of CgHOG1 in stress responses and glycerol overproduction of *Candida glycerinogenes*. Current Microbiology.

[CR24] Kassambara A (2017). Ggpubr:“ggplot2” based publication ready plots.

[CR25] Koutsovoulos G, Kumar S, Laetsch DR, Stevens L, Daub J, Conlon C, Maroon H, Thomas F, Aboobaker AA, Blaxter M (2016). No evidence for extensive horizontal gene transfer in the genome of the tardigrade Hypsibius dujardini. Proceedings of the National Academy of Sciences of the United States of America.

[CR26] Kullman B, Tamm H, Kullman K (2005). Fungal Genome Size Database.

[CR27] Kuske Cheryl R., Hesse Cedar N., Challacombe Jean F., Cullen Daniel, Herr Joshua R., Mueller Rebecca C., Tsang Adrian, Vilgalys Rytas (2015). Prospects and challenges for fungal metatranscriptomics of complex communities. Fungal Ecology.

[CR28] Kwon-Chung KJ, Bennett JE, Wickes BL, Meyer W, Cuomo CA, Wollenburg KR, Bicanic TA, Castaneda E, Chang YC, Chen J, Cogliati M, Dromer F, Ellis D, Filler SG, Fisher MC, Harrison TS, Holland SM, Kohno S, Kronstad JW, Lazera M, Levitz SM, Lionakis MS, May RC, Ngamskulrongroj P, Pappas PG, Perfect JR, Rickerts V, Sorrell TC, Walsh TJ, Williamson PR, Xu J, Zelazny AM, Casadevall A (2017) The case for adopting the "species complex" nomenclature for the etiologic agents of Cryptococcosis. *mSphere* 2(1). 10.1128/mSphere.00357-16 10.1128/mSphere.00357-16 PMC5227069 28101535

[CR29] Kwon-Chung Kyung J., Boekhout Teun, Fell Jack W., Diaz Mara (2002). (1557) Proposal to conserve the name Cryptococcus gattii against C. hondurianus and C. bacillisporus (Basidiomycota, Hymenomycetes, Tremellomycetidae ). TAXON.

[CR30] Langmead B, Salzberg SL (2012). Fast gapped-read alignment with bowtie 2. Nature Methods.

[CR31] Li B, Dewey CN (2011) RSEM: accurate transcript quantification from RNA-Seq data with or without a reference genome. BMC Bioinformatics 12:323. doi:10.1186/1471-2105-12-323 10.1186/1471-2105-12-323 PMC3163565 21816040

[CR32] Li X, Shen X, Chen X, Xiang D, Murphy RW, Shen Y (2018) Detection of potential problematic *Cytb* gene sequences of fishes in GenBank. Frontiers in Genetics 9:30. doi:10.3389/fgene.2018.00030 10.3389/fgene.2018.00030 PMC5808227 29467794

[CR33] Liao HL, Chen Y, Bruns TD, Peay KG, Taylor JW, Branco S, Talbot JM, Vilgalys R (2014). Metatranscriptomic analysis of ectomycorrhizal roots reveals genes associated with *Piloderma-Pinus* symbiosis: improved methodologies for assessing gene expression in situ. Environmental Microbiology.

[CR34] Loftus BJ, Fung E, Roncaglia P, Rowley D, Amedeo P, Bruno D, Vamathevan J, Miranda M, Anderson IJ, Fraser JA, Allen JE, Bosdet IE, Brent MR, Chiu R, Doering TL, Donlin MJ, D'Souza CA, Fox DS, Grinberg V, Fu J, Fukushima M, Haas BJ, Huang JC, Janbon G, Jones SJ, Koo HL, Krzywinski MI, Kwon-Chung JK, Lengeler KB, Maiti R, Marra MA, Marra RE, Mathewson CA, Mitchell TG, Pertea M, Riggs FR, Salzberg SL, Schein JE, Shvartsbeyn A, Shin H, Shumway M, Specht CA, Suh BB, Tenney A, Utterback TR, Wickes BL, Wortman JR, Wye NH, Kronstad JW, Lodge JK, Heitman J, Davis RW, Fraser CM, Hyman RW (2005). The genome of the basidiomycetous yeast and human pathogen *Cryptococcus neoformans*. Science.

[CR35] Marcelino VR, Verbruggen H (2016) Multi-marker metabarcoding of coral skeletons reveals a rich microbiome and diverse evolutionary origins of endolithic algae. Scientific Reports 6:31508 10.1038/srep31508 10.1038/srep31508 PMC4992875 27545322

[CR36] Martin-Laurent F, Philippot L, Hallet S, Chaussod R, Germon JC, Soulas G, Catroux G (2001). DNA extraction from soils: old bias for new microbial diversity analysis methods. Applied and Environmental Microbiology.

[CR37] McGuire KL, Payne SG, Palmer MI, Gillikin CM, Keefe D, Kim SJ, Gedallovich SM, Discenza J, Rangamannar R, Koshner JA, Massmann AL, Orazi G, Essene A, Leff JW, Fierer N (2013). Digging the new York City skyline: soil fungal communities in green roofs and city parks. PLoS One.

[CR38] Meyer W, Aanensen DM, Boekhout T, Cogliati M, Diaz MR, Esposto MC, Fisher M, Gilgado F, Hagen F, Kaocharoen S, Litvintseva AP, Mitchell TG, Simwami SP, Trilles L, Viviani MA, Kwon-Chung J (2009). Consensus multi-locus sequence typing scheme for *Cryptococcus neoformans* and *Cryptococcus gattii*. Medical Mycology.

[CR39] Munoz JF, Gade L, Chow NA, Loparev VN, Juieng P, Berkow EL, Farrer RA, Litvintseva AP, Cuomo CA (2018). Genomic insights into multidrug-resistance, mating and virulence in *Candida auris* and related emerging species. Nature Communications.

[CR40] Ngamskulrungroj P, Gilgado F, Faganello J, Litvintseva AP, Leal AL, Tsui KM, Mitchell TG, Vainstein MH, Meyer W (2009). Genetic diversity of the *Cryptococcus* species complex suggests that *Cryptococcus gattii* deserves to have varieties. PLoS One.

[CR41] Nilsson RH, Anslan S, Bahram M, Wurzbacher C, Baldrian P, Tedersoo L (2019). Mycobiome diversity: high-throughput sequencing and identification of fungi. Nature Reviews. Microbiology.

[CR42] Nilsson R. Henrik, Kristiansson Erik, Ryberg Martin, Hallenberg Nils, Larsson Karl-Henrik (2008). IntraspecificITSVariability in the KingdomFungias Expressed in the International Sequence Databases and Its Implications for Molecular Species Identification. Evolutionary Bioinformatics.

[CR43] Olm MR, West PT, Brooks B, Firek BA, Baker R, Morowitz MJ, Banfield JF (2019). Genome-resolved metagenomics of eukaryotic populations during early colonization of premature infants and in hospital rooms. Microbiome.

[CR44] Pruitt KD, Tatusova T, Maglott DR (2007). NCBI reference sequences (RefSeq): a curated non-redundant sequence database of genomes, transcripts and proteins. Nucleic Acids Research.

[CR45] Quince C, Walker AW, Simpson JT, Loman NJ, Segata N (2017). Shotgun metagenomics, from sampling to analysis. Nature Biotechnology.

[CR46] Reck M, Tomasch J, Deng Z, Jarek M, Husemann P, Wagner-Dobler I, Consortium C (2015) Stool metatranscriptomics: a technical guideline for mRNA stabilisation and isolation. BMC Genomics 16:494. 10.1186/s12864-015-1694-y 10.1186/s12864-015-1694-y PMC4490624 26140923

[CR47] Rizzetto L, Giovannini G, Bromley M, Bowyer P, Romani L, Cavalieri D (2013). Strain dependent variation of immune responses to *A. fumigatus*: definition of pathogenic species. PLoS One.

[CR48] Robert V, Vu D, Amor AB, van de Wiele N, Brouwer C, Jabas B, Szoke S, Dridi A, Triki M, Ben Daoud S, Chouchen O, Vaas L, de Cock A, Stalpers JA, Stalpers D, Verkley GJ, Groenewald M, Dos Santos FB, Stegehuis G, Li W, Wu L, Zhang R, Ma J, Zhou M, Gorjon SP, Eurwilaichitr L, Ingsriswang S, Hansen K, Schoch C, Robbertse B, Irinyi L, Meyer W, Cardinali G, Hawksworth DL, Taylor JW, Crous PW (2013). MycoBank gearing up for new horizons. IMA Fungus.

[CR49] Salter SJ, Cox MJ, Turek EM, Calus ST, Cookson WO, Moffatt MF, Turner P, Parkhill J, Loman NJ, Walker AW (2014) Reagent and laboratory contamination can critically impact sequence-based microbiome analyses. BMC Biology 12:87. 10.1186/s12915-014-0087-z 10.1186/s12915-014-0087-z PMC4228153 25387460

[CR50] Schmidt Philipp-André, Bálint Miklós, Greshake Bastian, Bandow Cornelia, Römbke Jörg, Schmitt Imke (2013). Illumina metabarcoding of a soil fungal community. Soil Biology and Biochemistry.

[CR51] Schmieder R, Edwards R (2011). Quality control and preprocessing of metagenomic datasets. Bioinformatics.

[CR52] Sczyrba A, Hofmann P, Belmann P, Koslicki D, Janssen S, Droge J, Gregor I, Majda S, Fiedler J, Dahms E, Bremges A, Fritz A, Garrido-Oter R, Jorgensen TS, Shapiro N, Blood PD, Gurevich A, Bai Y, Turaev D, DeMaere MZ, Chikhi R, Nagarajan N, Quince C, Meyer F, Balvociute M, Hansen LH, Sorensen SJ, Chia BKH, Denis B, Froula JL, Wang Z, Egan R, Don Kang D, Cook JJ, Deltel C, Beckstette M, Lemaitre C, Peterlongo P, Rizk G, Lavenier D, Wu YW, Singer SW, Jain C, Strous M, Klingenberg H, Meinicke P, Barton MD, Lingner T, Lin HH, Liao YC, Silva GGZ, Cuevas DA, Edwards RA, Saha S, Piro VC, Renard BY, Pop M, Klenk HP, Goker M, Kyrpides NC, Woyke T, Vorholt JA, Schulze-Lefert P, Rubin EM, Darling AE, Rattei T, McHardy AC (2017). Critical assessment of metagenome interpretation-a benchmark of metagenomics software. Nature Methods.

[CR53] Seed PC (2014). The human mycobiome. Cold Spring Harbor Perspectives in Medicine.

[CR54] Shakya M, Quince C, Campbell JH, Yang ZK, Schadt CW, Podar M (2013). Comparative metagenomic and rRNA microbial diversity characterization using archaeal and bacterial synthetic communities. Environmental Microbiology.

[CR55] Shi M, Lin XD, Tian JH, Chen LJ, Chen X, Li CX, Qin XC, Li J, Cao JP, Eden JS, Buchmann J, Wang W, Xu J, Holmes EC, Zhang YZ (2016). Redefining the invertebrate RNA virosphere. Nature.

[CR56] Shi M, Neville P, Nicholson J, Eden JS, Imrie A, Holmes EC (2017) High-resolution metatranscriptomics reveals the ecological dynamics of mosquito-associated rna viruses in Western Australia. Journal of Virology 91(17):e00680–17. 10.1128/JVI.00680-17 10.1128/JVI.00680-17 PMC5553174 28637756

[CR57] Soll DR, Staebell M, Langtimm C, Pfaller M, Hicks J, Rao TV (1988). Multiple *Candida* strains in the course of a single systemic infection. Journal of Clinical Microbiology.

[CR58] Strong MJ, Xu G, Morici L, Splinter Bon-Durant S, Baddoo M, Lin Z, Fewell C, Taylor CM, Flemington EK (2014). Microbial contamination in next generation sequencing: implications for sequence-based analysis of clinical samples. PLoS Pathogens.

[CR59] Tedersoo L, Anslan S, Bahram M, Põlme S, Riit T, Liiv I, Kõljalg U, Kisand V, Nilsson H, Hildebrand F, Bork P, Abarenkov K (2015). Shotgun metagenomes and multiple primer pair-barcode combinations of amplicons reveal biases in metabarcoding analyses of fungi. MycoKeys.

[CR60] Wang Z, Gerstein M, Snyder M (2009). RNA-Seq: a revolutionary tool for transcriptomics. Nature Reviews. Genetics.

[CR61] Wang Z, Zhuge J, Fang H (1999). A new osmotolerant and glycerol-highly-producing species - *Candida glycerolgenesis* Zhuge sp. nov. Acta Microbiologica Sinica.

[CR62] Wille M, Eden JS, Shi M, Klaassen M, Hurt AC, Holmes EC (2018). Virus-virus interactions and host ecology are associated with RNA virome structure in wild birds. Molecular Ecology.

[CR63] Yahr Rebecca, Schoch Conrad L., Dentinger Bryn T. M. (2016). Scaling up discovery of hidden diversity in fungi: impacts of barcoding approaches. Philosophical Transactions of the Royal Society B: Biological Sciences.

[CR64] Yamamoto N, Dannemiller KC, Bibby K, Peccia J (2014). Identification accuracy and diversity reproducibility associated with internal transcribed spacer-based fungal taxonomic library preparation. Environmental Microbiology.

[CR65] Zhang YZ, Shi M, Holmes EC (2018). Using metagenomics to characterize an expanding virosphere. Cell.

