# Supplementary material for: Metatranscriptomics as a tool to identify fungal species and subspecies in mixed communities – a proof of concept under laboratory conditions
Source: IMA Fungus. 2019 Aug 8;10:12. doi: 10.1186/s43008-019-0012-8 (PMC7184889; doi:10.1186/s43008-019-0012-8)
Supplement: Supplementary file 2 — Figure indicating that no correlation between abundance of transcripts (depth) and genome size (A) or protein count (B) can be observed. (PDF 881 kb) [file 43008_2019_12_MOESM2_ESM.pdf]

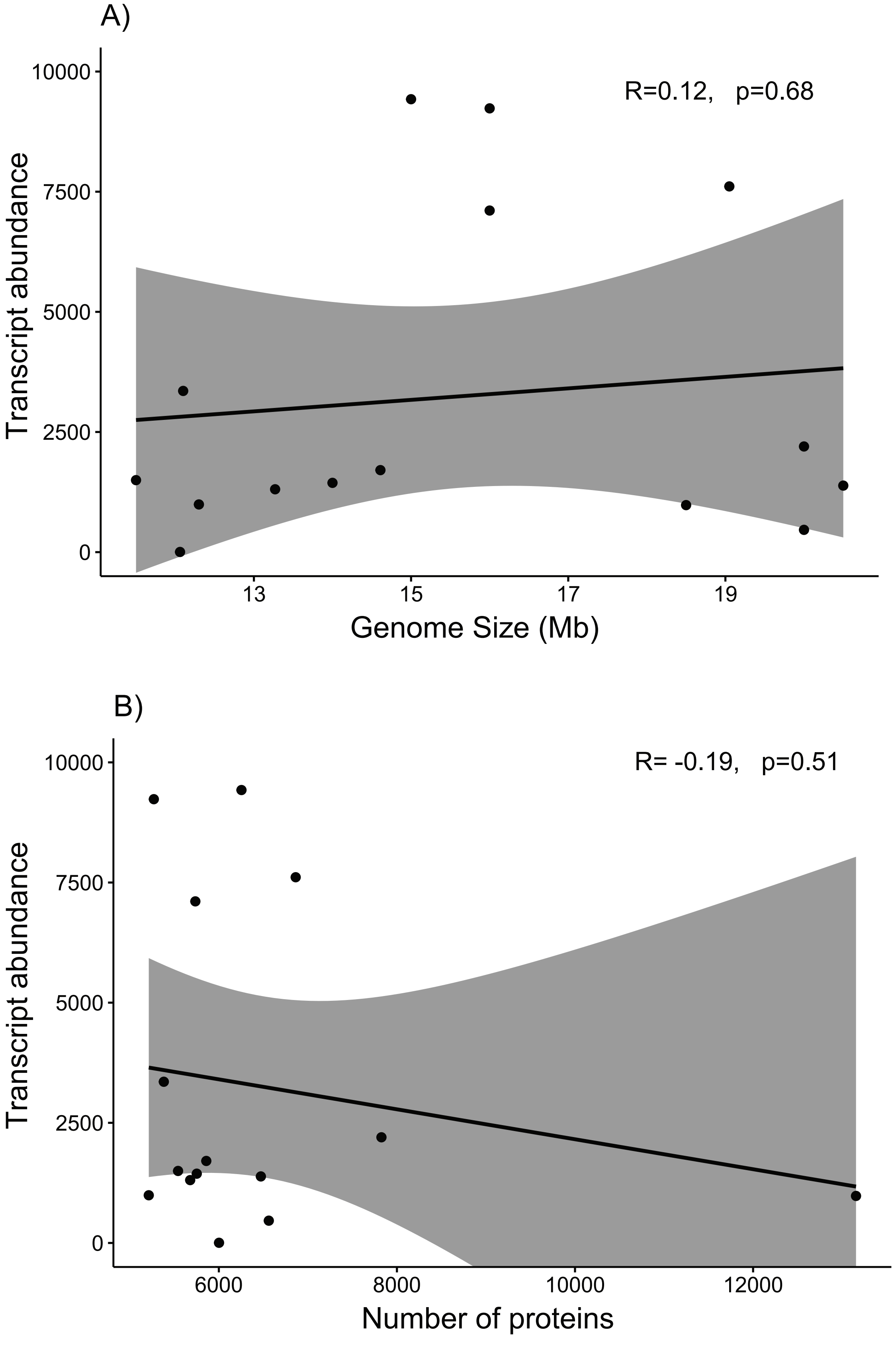

Additional file 2. No correlation between abundance of transcripts (depth) and genome size (A) or protein count (B) was observed. Each dot represents a fungal species included in the mock community. Pearson's correlation R and p values are given. Shaded areas indicate 95% confidence interval.
